# Impact of the COVID-19 Pandemic on Life Space Extent and Apathy: A Comparison of Competitive Japanese Swimmers with and without Disabilities

**DOI:** 10.3390/ijerph18105106

**Published:** 2021-05-12

**Authors:** Kazuki Kaneda, Noriaki Maeda, Yuta Suzuki, Kazuki Fukui, Yukio Urabe

**Affiliations:** 1 Graduate School of Biomedical and Health Sciences, Hiroshima University, Hiroshima 734-8553, Japan; kazuki-kaneda@hiroshima-u.ac.jp (K.K.); norimmi@hiroshima-u.ac.jp (N.M.); kazuki-fukui@hiroshima-u.ac.jp (K.F.); 2 Department of Rehabilitation, Matterhorn Rehabilitation Hospital, Hiroshima 737-0046, Japan; yt.suzuki28@gmail.com

**Keywords:** the coronavirus disease COVID-19, female athlete, physical impairment, para-sport, swimmer, life space, mental health

## Abstract

Changes in the daily lives and mental health of people with disabilities due to the coronavirus disease (COVID-19) pandemic have not been reported. The Japanese government closed public facilities, including swimming pools, during the first wave of COVID-19, and many competitive swimmers lost their places of activities. This study aimed to investigate the impact of the COVID-19 pandemic on life space and apathy among swimmers and investigated differences in the impact on swimmers with and without disabilities. A total of 39 competitive swimmers participated in this study, including 11 male and nine female swimmers with disabilities (swimmers with disabilities = para-swimmers), and e11 male and eight female swimmers without disabilities. Baseline and follow-up web-based questionnaire surveys were conducted, and changes in life space and apathy scale (AS) were assessed. Female para-swimmers showed significantly lower apathy than female able-bodied subjects (para, during; 16.0 ± 1.9; after, 12.8 ± 3.2; non-disabled; during, 10.5 ± 4.4; after, 10.6 ± 4.8; *p* < 0.05). Female swimmers with disabilities may be more likely to experience worsening mental health due to changes in their lifestyle.

## 1. Introduction

The COVID-19 outbreak was first reported in December 2019 [1] and has since spread to many countries globally. On 11 March 2020, the World Health Organization (WHO) declared it as a global pandemic [2], and on 16 April 2020, the Japanese government declared a nationwide state of emergency [3]. The Japanese government instructed citizens to refrain from non-essential outings and asked commercial facilities to close [4]. These countermeasures were supposed to protect against the spread of infection, but the effects changed living circumstances and narrowed the life space [5,6]. 

It is known that a decrease in life space and physical activity due to reduced mobility status can lower an individual’s general mental health [7], often causing people to fall into temporary depressive states and decrease apathy [8]. In addition, it has been found that people with chronic disabilities tend to have smaller life spaces and are more prone to mental stress [9]. Therefore, it is necessary to pay more attention to the changes in mental health associated with the narrow lives of people with disabilities [10]. Alternatively, exercise and sports activities play a preventive role against mental health exacerbations, so it is extremely important to exercise and participate in sports even during a state of emergency [11]. As part of the declaration of the state of emergency due to the effects of COVID-19, “stay at home” may also affect those who habitually exercise and play sports [12]. Due to the declaration of a state of emergency, it was impossible to play sports using the facilities, and except for jogging, most people were forced to limit themselves to exercising at home [13]. Competitive swimming, in particular, is a sport that cannot be practiced without the availability of specific facilities with a pool. There is an option to practice in the ocean or lake, but it is in fact difficult in April due to the climate. Therefore, during the declaration, the mobility status of life may be sharply reduced, and life space may become narrower for all the swimmers [6]. In addition, for swimmers with disabilities (swimmers with disabilities = para-swimmers), participation in sports plays an important role in improving their physical and mental health and enhancing their daily life skills. The reasons why people with disabilities participate in sports are diverse and include functional recovery, health maintenance, making friends, and achieving social participation [14]. Therefore, for people with disabilities, maintenance of their life spaces affects their physical activity and is likely important for the maintenance of their mental health. Among mental health, apathy is defined as “a lack or a reduction of feeling, emotion, interest, or concern, not attributable to a diminished level of consciousness.” [15] Thus, maintaining apathy plays an important role in maintaining motivation and interest.

However, from our search of previous studies, it is unclear whether the closure of pools affected the life space and apathy of swimmers. In particular, para-swimmers may be more affected, but no studies have investigated changes even in the mental health and apathy in para-swimmers. Therefore, the purpose of this study was to investigate whether the apathy of para-swimmers changed due to environmental changes caused by COVID-19. We hypothesized that the life space would be smaller during the state of emergency than after it was lifted, and that the impact on apathy would have been greater for para-swimmers than for able-bodied swimmers.

## 2. Materials and Methods

This study was conducted with the approval of the Chushikoku-Shikoku Para Swimming Federation and three swimming clubs in the Chugoku and Shikoku regions that were asked to participate in the web-based survey. Although it was difficult to conduct face-to-face surveys while the state of emergency was declared, many studies have conducted web-based surveys, which have been reported to be highly reliable [16]. In this study, we used Google Forms, which is commonly used in research papers [17]. 

All participants signed an informed consent form approved by the Ethics Committee for Epidemiology of Hiroshima University (approval ID: E-3544).

### 2.1. Participants and Survey Procedures

A total of 60 subjects (30 male and female able-bodied swimmers and 30 male and female Para-swimmers) aged 16 to 80 years participated in this study; and their sex and age were matched. The common inclusion criteria were swimmers aged 16 years or older and practiced at least once a week, and the exclusion criteria were swimmers aged 15 years or younger and practiced only once every 2–3 weeks.

The first online questionnaire was conducted between 16 April to 24 May 2020, during the state of emergency and assessed the baseline characteristics of COVID-19. During this period, the COVID-19 pandemic information and Life-Space Assessment and apathy assessment were conducted. In addition, the evaluation of living space before the emergency was also conducted; this was used as the baseline. Simultaneously, the para-swimmers were asked to name their disabilities and class status. The second questionnaire was conducted from 25 May to 21 June 2020, after the state of emergency had been lifted, and their life-space assessment and apathy were investigated and compared.

### 2.2. Demographics

In terms of basic information, all participants were asked to provide their age, height, weight, body-mass index (BMI), swimming competitive history, and number of practice sessions per week. Para-swimming participants were asked additional questions about their disability diagnosis name and para-swimming classification status as basic information.

There are 10 types of functional disabilities that meet the World Para Swimming (WPS) requirements: hypermobility, ataxia, athetosis, limb amputation, muscle weakness, limited range of motion, dwarfism, leg length difference, visual impairment, and intellectual disability, with medically confirmed findings. Para-swimmers are classified into physical disability classes (S1 to S10), visual disability classes (S11 to S13), intellectual disability classes (S14), and hearing disability classes (S15) to ensure fairness [18]. In the physical disability and visual disability classification, the lower the number, the greater the degree of disability.

### 2.3. Life-Space Assessment

The life-space assessment was conducted using the Japanese version of the Life-Space Assessment scale (LSA) Japanese Edition, which was adapted by Harada et al. [19], who based the instrument on the LSA developed by Baker et al. [20] and translated it into Japanese. The LSA has been proven to be a reliable assessment instrument that investigated life space [19,20,21]. The LSA is an index that evaluated the range of movement, frequency of movement, and degree of independence in each living space based on the concept of mobility in living spaces. The life space was divided into six categories: bedroom, inside the home, outside the home, near the home (within 800 m), inside the town (more than 800 m to 16 km), and outside the town (more than 16 km). The final score was calculated as the total score of each life space. The LSA score was on a 120-point scale, and higher scores indicated a wider range of activities.

### 2.4. Japanese Apathy Scale

The Japanese Apathy Scale for Japanese (JAS) was used to assess apathy. The JAS was developed by translating the Apathy Scale [22] and standardizing it for the Japanese population. The apathy scale is an excellent measure of motivation and interest, and is also used as a survey item for mental health [22,23,24]. The questionnaire consisted of questions such as: (1) Do you want to learn new things, (2) Do you have any interests, (3) Are you interested in your health, (4) Are you able to put your mind to things, and (5) Do you always want to do something, for example. All 14 questions were answered on a four-point scale from 0 to 3, and a score of 16 or higher indicated low motivation [22].

### 2.5. Statistical Analysis

The normality of the subjects’ demographic characteristics (age, BMI, and competition history) was confirmed using the Shapiro-Wilk test. Since there was normality, para-swimmers and able-bodied swimmers were compared by gender using an uncorrelated *t*-test. To analyze the changes in LSA and JAS through the COVID-19 pandemic, a two-way repeated-measures ANOVA with group as a between-participants factor and time as a within-participant factor was used. For LSA, the three factors were adapted for the within-participant factor: baseline, during, and at follow-up, while for JAS, the two factors were applied during follow-up. In the case of a significant interaction, an unpaired *t*-test was used to compare the para-swimmers and able-bodied swimmers. One-way analysis of variance and multiple comparison tests were used for post-hoc tests when a significant main effect was detected, and corresponding *t*-tests between groups were used when a significant interaction was detected. The statistical analyses were performed with EZR (Saitama Medical Center, Jichi Medical University, Saitama, Japan), which is a graphical user interface for R (The R Foundation for Statistical Computing, Vienna, Austria) EZR is a statistical software that extends the functionality of R and R Commander, and the reliability of its statistical analysis results has been confirmed [25]. The level of significance for all analyses was set at *p* < 0.05.

## 3. Results

**Figure 1:**
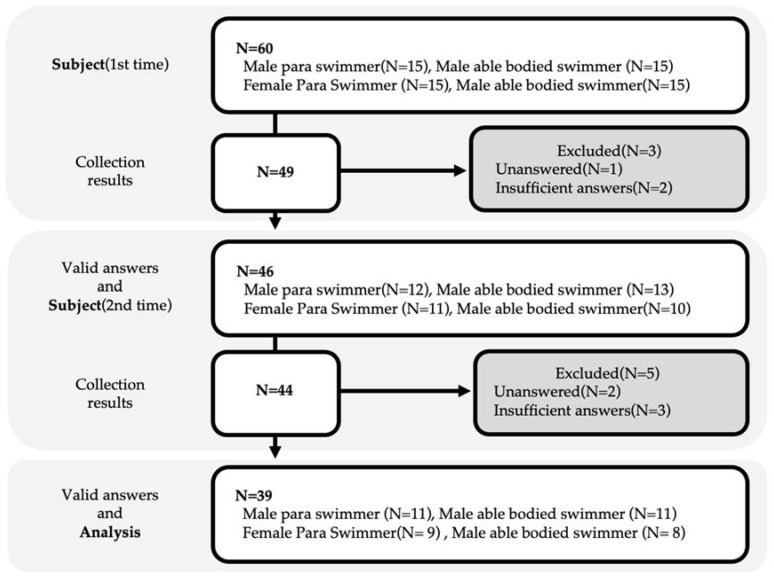
Flowchart with the inclusion/exclusion of participants.

The flowchart of the survey is shown in Figure 1. Forty-nine people responded to the first questionnaire, of which three were excluded, and hence, 46 (76.7%) participants were included. The second questionnaire was administered to the 46 valid respondents of the first questionnaire, and we received 44 responses, of which five were excluded, and 39 were finally included in the analysis. Of these, 11 (28.2%) were male para-swimmers, nine (23.0%) were female para-swimmers, 11 (28.2%) were male able-bodied swimmers, and eight (20.5%) were female able-bodied swimmers. Those who were excluded had inadequate or inappropriate scores.

Table 1 shows the demographics of the subjects and the characteristics of the Para-Swimmer. Though there was a significant difference in height and weight between male para-swimmers and healthy subjects (*p* < 0.01), there was no significant difference in BMI. Other than that, there were no significant differences in the demographics of the para-swimmers and healthy swimmers in all parameters.

### 3.1. Comparison of LSA Baseline, during, and Follow-Up of the Declaration of a State of Emergency

**Figure 2:**
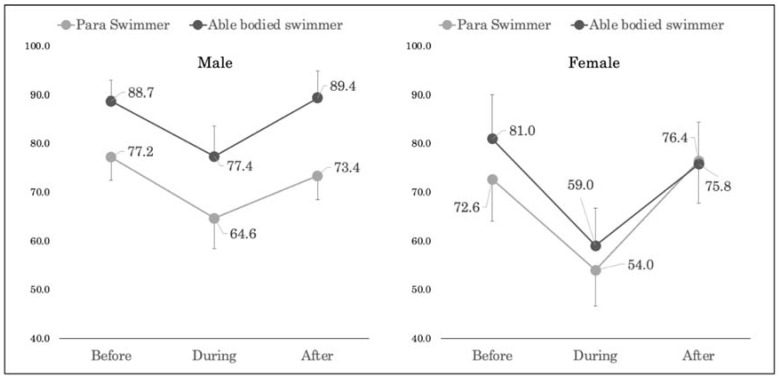
Comparison of LSA baseline, during, and follow-up the declaration of a state of emergency.

The changes in LSA at baseline, during, and at follow-up after the state of emergency are shown in Figure 2. 

The LSA of male para-swimmers showed an 18% decrease from baseline to during the state of emergency and a 12% increase from during to follow-up after the state of emergency. Female para-swimmers showed a 26% decrease from baseline to during the state of emergency and a 30% increase from during to follow-up after the state of emergency. Male able-bodied swimmers showed a 13% decrease from baseline to during the state of emergency and a 15% increase from during to follow-up after the state of emergency. Female able-bodied swimmers showed a 28% decrease from baseline to during the state of emergency and a 23% increase from during to follow-up after state of emergency. 

Two-way ANOVA showed that there was no significant interaction between the male and female groups in the LSA. There was no significant main effect between groups in the female group, but there was a significant effect in the male group.

The LAS during and at follow-up declaration within the male and female groups were analyzed using a two-way analysis of variance. A one-way analysis of variance was used as a post-hoc test, but no significant difference in time was observed. In the female group, there was no significant main effect between the groups or time, while in the male group, there was a significant main effect among the groups. 

### 3.2. Comparison of the JAS during and Follow-Up Post the State of Emergency Declaration

A comparison of the JAS during and after the declaration of the state of emergency is shown in Table 2. 

The male para-swimmers scored 12.5 ± 8.1 during the declaration and 11.2 ± 5.9 at follow-up after the declaration was lifted, a decrease of 11%. The female para-swimmers scored 16.0 ± 1.9 points during the declaration and 12.8 ± 3.2 points at follow-up after, a decrease of 20%. The male able-bodied swimmers scored 12.3 ± 6.4 points during and 8.8 ± 5.2 points at follow-up, a decrease of 29%. The female able-bodied swimmers were second with 10.5 ± 4.4 points during the declaration and 10.6 ± 4.8 points at follow-up, which showed almost no change. There was no significant interaction between male para-swimmers and able-bodied swimmers (F = 0.34, *p* = 0.56), and no significant main effect between groups or at the time of the study (F = 0.45, *p* = 0.51; at the time of the study, F = 2.28, *p* = 0.14). Alternatively, there was a significant interaction between female para-swimmers and able-bodied swimmers (F = 5.45, *p* < 0.05) and a significant main effect both between groups and at the time of the study (F = 0.462, *p* < 0.05; time of the study: F = 4.67, *p* < 0.05). In the corresponding *t*-test, the JAS of para-swimmers showed significantly lower values (*p* < 0.05), and in the non-corresponding *t*-test, para-swimmers showed significantly higher values in the JAS during declaration (*p* < 0.01).

## 4. Discussion

This study investigated the changes in the life-space and apathy of regional para-swimmers within a specific period who lost the opportunity to participate in competitive sports due to the declaration of a state of emergency related to COVID-19 in Japan. The novelty of this study lies in the fact that we included para-swimmers who had exercise habits in the study. Due to the declaration of a state of emergency, participation in sports, which has a high priority in daily life, became restricted and caused major changes in the participants’ daily lives. However, this change was believed to have decreased the apathy of only the participating female para-swimmers during the state of the emergency, and their apathy showed improvement a month after the declaration was lifted.

The LSA of all subjects in this study decreased during the declaration of a state of emergency, and improved at follow-up after the state of emergency was lifted. It was said that the number of steps decreased and the sitting time increased with the narrowing of the life space [26]. The increase in sitting time is closely related to the decrease in physical and psychological health and apathy [27], and the effects of the declaration of a state of emergency or the lockdown have caused the deterioration of health and apathy worldwide [28]. Alternatively, after the declaration of a state of emergency or the lifting of a lockdown, it is possible that the amount of physical activity and mental health that declined during the period may improve. In the present study, male para-swimmers, healthy swimmers, and female para-swimmers showed a decrease in the JAS and an improvement in apathy following the lifting of the state of emergency declaration. This may be due to the fact that the expansion of the life space increased the amount of physical activity and led to the improvement of the JAS. In female able-bodied swimmers, there was no significant change in the JAS during or after the declaration of the state of emergency. While many studies have reported an association between increased physical activity and improved mental health [29], female able-bodied swimmers may have been less likely to experience a decline in mental health because they fulfill their daily activities by performing household chores [30]. One of the possible reasons for the lack of change in JAS was that the required daily living activities (more than 30 min at an intensity of more than three metabolic equivalent of tasks (METs), more than twice a week) were maintained by housework, but since it is unclear how much housework was actually performed in this study, it will be necessary to further investigate the status of housework during the declaration of the state of emergency.

What is noteworthy in the present results is that only female para-swimmers showed a decrease in apathy, as their JAS during the declaration of a state of emergency was higher than 16 points. During the declaration of a state of emergency, sports and exercise facilities such as swimming pools were shut down, and all subjects, regardless of whether they were para-athletes, healthy athletes, or men and women, lost the opportunity to swim. In a previous study, aerobic exercise such as running for depressed patients and those with weakened apathy had the same effect as psychotherapy [10], which indicated that aerobic exercise had an effect on mental health. This suggests that while able-bodied swimmers could easily substitute aerobic exercise and training for swimming, para-swimmers may not have been able to adequately perform aerobic exercise and training on land due to their disease-related disabilities. Women and people with disabilities are more likely to be subjected to negative attitudes by those around them, which has been considered a problem [31]. In addition, women and the elderly are more vulnerable to changes in the social environment [32], such as epidemics of infection, and are more likely to show psychological distress, and among people with disabilities, women are more at risk of social isolation than men [33]. These findings suggest that female para-swimmers, in particular, may have been significantly affected by the narrowing of their living space due to the declaration of the state of emergency, and may have shown a decrease in apathy.

The present study showed that COVID-19-induced changes in living conditions resulted in decreased apathy in able-bodied swimmers and para-swimmers, but the effects were particularly significant in women with disabilities. Para-swimmers may not be able to perform aerobic exercise on land, which can be substituted for swimming, and are also limited in what they can do at home. Therefore, it is important to promote the management of sports venues at the local level [34], create alternative opportunities to exercise by planning exercises that can be performed on the spot, regardless of location or situation, and consider psychological health from the aspect of gender and disability.

### Limitations and Strengths of the Study

The limitations of this study are as follows: First, the study area and target population were limited, and the impact may be different compared to other areas. Second, the number of evaluation items was limited. Third, the target of the survey was small in size. However, this web-based survey required speed and simplicity because it was conducted via the Internet for few para-athletes. The JAS before the declaration of the state of emergency was not measured because of the possibility of recall bias, but if it had been measured, this study would be confident. The evaluation items were based on evaluation indices that have been used in several studies, and their reliability has been confirmed. In the future, we would like to increase the number of items for quality of life (QOL) and physical activity to evaluate those in more detail. In addition, since there are no studies focusing on para-athletes, we believe that the results of this study will help us plan countermeasures in response to possible changes in the situation.

## 5. Conclusions

This study investigated the impact of changes in living conditions during and after the declaration of a state of emergency on the life space and mental health of swimmers. In terms of the life space, all subjects in this study showed a decrease in LSA during the declaration of the state of emergency and an increase in follow-up. The JAS of male and female para-swimmers and male able-bodied swimmers improved after the state of emergency was lifted, while female able-bodied swimmers showed no change. Only female para-swimmers showed a decrease in apathy, as their JAS during the state of emergency was higher than 16 points. In the future, we would like to prevent and improve the mental health of female para-athletes due to environmental changes by creating opportunities for exercise that can be done on the spot and examining measures that take psychological health into consideration from the aspects of gender and disability.

## Figures and Tables

**Table 1:** Participants’ demographics and baseline (*N* = 39).

Variable	Male Swimmers			Female Swimmers		
	Para (*N* = 11)	Able-Bodied (*N* = 11)	*p*	Para (*N* = 9)	Able-Bodied (*N* = 8)	*p*
Age (year)	44 ± 17.2	46.2 ± 22.2	0.77	31 ± 13.2	38.9 ± 23.5	0.42
Height (cm)	163.8 ± 7.1	175.3 ± 2.7	0.00	158.9 ± 4.1	156.4 ± 4.4	0.27
Weight (kg)	61.1 ± 9.9	70.7 ± 4.5	0.01	54.1 ± 4.7	53.8 ± 4.7	0.88
BMI (kg/m^2^)	23 ± 3.1	23 ± 1.3	0.76	21 ± 1.5	21.9 ± 1	0.45
Competition history (years)	23 ± 11.9	35.6 ± 20	0.06	15 ± 13.5	30.4 ± 19.8	0.09
Regularity of practice (times/week)	2.7 ± 1.8	3.8 ± 2.9	0.32	4.8 ± 2.1	5.1 ± 2.3	0.76
Social status						
Student	2 (18.2)	4 (36.4)		3 (33.3)	3 (37.5)	
Company employee	7 (63.6)	3 (27.4)		5 (55.6)	3 (37.5)	
Self-employed	−	1 (9.0)		−	−	
Unemployed	2 (12.8)	3 (27.4)		1 (11.1)	2 (25.0)	
Living Alone	4 (36.4)	0 (0.0)		4 (44.4)	1 (12.5)	
Para Classification State						
S4	−	−		1 (11.1)	−	
S6	4 (36.4)	−		1 (11.1)	−	
S7	−	−		1 (11.1)	−	
S8	1 (9.0)	−		1 (11.1)	−	
S9	3 (27.4)	−		3 (33.3)	−	
S10	2 (12.8)	−		1 (11.1)	−	
S11	−	−		1 (11.1)	−	
S12	1 (33.2)	−		−	−	
Type of disability						
Cerebral palsy	3 (27.4)	−		3 (33.3)	−	
Amputation/Deficit	3 (27.4)	−		2 (22.2)	−	
Visual impairment	1 (9.0)	−		1 (11.1)	−	
Hemiplegia	2 (12.8)	−		1 (11.1)	−	
Spinal cord injury	1 (9.0)	−		−	−	
Osteoarthritis	1 (9.0)	−		1 (11.1)	−	
Multiple sclerosis	−	−		1 (11.1)	−	

Mean ± SD, *N* (%), BMI: Body-Mass Index, Non-responsive *t*-test (Age, BMI, Competition history).

**Table 2:** Changes in the JAS of male and female swimmers during and follow-up post the lifting of the emergency.

Variable	Para Swimmer Group			Able-Bodied Swimmer Group			Interaction Effect		Main Effect	
							(Group × Time)		(Time)	
							F Value	*p*-Value	F Value	*p*-Value
Male										
During	12.5	±	8.1	12.3	±	6.4	0.34	0.56	2.28	0.14
Follow-up	11.2	±	5.9	8.8	±	5.2				
Female										
During	16.0	±	1.9	10.5	±	4.4	5.45	<0.05	4.67	<0.05
Follow-up	12.8	±	3.2	10.6	±	4.8				

Mean ± SD, JAS: Japanese Apathy Scale. Significant differences between during and follow-up emergencies in JAS of the female para swimmer (*p* < 0.05).

## Data Availability

The data presented in this study are available on request from the corresponding author. The data are not publicly available due to restrictions of privacy.

## References

[1] Ren L.-L., Wang Y.-M., Wu Z.-Q., Xiang Z.-C., Guo L., Xu T., Jiang Y.-Z., Xiong Y., Li Y.-J., Li X.-W. (2020). Identification of a novel coronavirus causing severe pneumonia in human: A descriptive study. Chin. Med. J..

[2] Yanagawa A. (2020). About Outbreak of Pneumonia Patient Associated with New Coronavirus (1st Case).

[3] (2020). Timeline of WHO’s Response to COVID-19.

[4] (2020). Headquarters for the Control of New Coronavirus Infections.

[5] Mehta V. (2020). The new proxemics: COVID-19, social distancing, and sociable space. J. Urban Des..

[6] Suzuki Y., Maeda N., Hirado D., Shirakawa T., Urabe Y. (2020). Physical Activity Changes and Its Risk Factors among Community-Dwelling Japanese Older Adults during the COVID-19 Epidemic: Associations with Subjective Well-Being and Health-Related Quality of Life. Int. J. Environ. Res. Public Health.

[7] Lampinen P., Heikkinen E. (2003). Reduced Mobility and Physical Activity as Predictors of Depressive Symptoms among Commu-nity-Dwelling Older Adults: An Eight-Year Follow-Up Study. Aging Clin. Exp. Res..

[8] Stavrakakis N., De Jonge P., Ormel J., Oldehinkel A.J. (2012). Bidirectional Prospective Associations Between Physical Activity and Depressive Symptoms. The TRAILS Study. J. Adolesc. Health.

[9] Battalio S.L., Huffman S.E., Jensen M.P. (2020). Longitudinal associations between physical activity, anxiety, and depression in adults with long-term physical disabilities. Health Psychol..

[10] Tough H., Siegrist J., Fekete C. (2017). Social relationships, mental health and wellbeing in physical disability: A systematic review. BMC Public Health.

[11] Stathopoulou G., Powers M.B., Berry A.C., Smits J.A.J., Otto M.W. (2006). Exercise Interventions for Mental Health: A Quantitative and Qualitative Review. Clin. Psychol. Sci. Pract..

[12] Shepherd H., Evans T., Gupta S., McDonough M., Doyle-Baker P., Belton K., Karmali S., Pawer S., Hadly G., Pike I. (2021). The Impact of COVID-19 on High School Student-Athlete Experiences with Physical Activity, Mental Health, and Social Connection. Int. J. Environ. Res. Public Health.

[13] Maugeri G., Castrogiovanni P., Battaglia G., Pippi R., D’Agata V., Palma A., Di Rosa M., Musumeci G. (2020). The impact of physical activity on psychological health during Covid-19 pandemic in Italy. Heliyon.

[14] Ashton-Shaeffer C., Gibson H.J., Autry C.E., Hanson C.S. (2001). Meaning of Sport to Adults with Physical Disabilities: A Disability Sport Camp Experience. Sociol. Sport J..

[15] Marin R.S. (1991). Apathy: A neuropsychiatric syndrome. J. Neuropsychiatry Clin. Neurosci..

[16] Trespalacios J.H., Perkins R.A. (2016). Effects of Personalization and Invitation Email Length on Web-Based Survey Response Rates. TechTrends.

[17] Islam A., Barna S.D., Raihan H., Alam Khan N., Hossain T. (2020). Depression and anxiety among university students during the COVID-19 pandemic in Bangladesh: A web-based cross-sectional survey. PLoS ONE.

[18] (2018). World Para Swimming Classification Rules and Regulations.

[19] Harada K., Shimada H., Sawyer P., Asakawa Y., Nihei K., Kaneya S., Furuna T., Ishizaki T., Yasumura S. (2010). Life-space of community-dwelling older adults using preventive health care services in Japan and the validity of composite scoring methods for assessment. Nihon Koshu Eisei Zasshi.

[20] Baker P.S., Bodner E.V., Allman R.M. (2003). Measuring Life-Space Mobility in Community-Dwelling Older Adults. J. Am. Geriatr. Soc..

[21] Curcio C.L., Alvarado B.E., Gomez F., Guerra R., Guralnik J., Zunzunegui M.V. (2013). Life-Space Assessment Scale to Assess Mo-bility: Validation in Latin American Older Women and Men. Aging Clin. Exp. Res..

[22] Okada K., Kobayashi S., Aoki K., Suyama N., Yamaguchi S. (1998). Assessment of motivational less in poststroke patients using the Japanese version of Starkstein’s Apathy Scale. Jpn. J. Stroke.

[23] Starkstein S.E., Fedoroff J.P., Price T.R., Leiguarda R., Robinson R.G. (1993). Apathy following cerebrovascular lesions. Stroke.

[24] Yuen S.A., Patricia L., Matthew A.J.A., Kinan M., Masud H. (2017). Distinct Subtypes of Apathy Revealed by the Apathy Motiva-tion Index. PLoS ONE.

[25] Kanda Y. (2013). Investigation of the Freely Available Easy-To-Use Software ‘EZR’ for Medical Statistics. Bone Marrow Transplant..

[26] Tison G.H., Avram R., Kuhar P., Abreau S., Marcus G.M., Pletcher M.J., Olgin J.E. (2020). Worldwide Effect of COVID-19 on Physical Activity: A Descriptive Study. Ann. Intern. Med..

[27] Kar G., Hedge A. (2020). Effects of a sit-stand-walk intervention on musculoskeletal discomfort, productivity, and perceived physical and mental fatigue, for computer-based work. Int. J. Ind. Ergon..

[28] Woods J.A., Hutchinson N.T., Powers S.K., Roberts W.O., Gomez-Cabrera M.C., Radak Z., Berkes I., Boros A., Boldogh I., Leeuwenburgh C. (2020). The COVID-19 pandemic and physical activity. Sports Med. Health Sci..

[29] Augestad L.B., Slettemoen R.P., Flanders W.D. (2008). Physical Activity and Depressive Symptoms Among Norwegian Adults Aged 20–50. Public Health Nurs..

[30] Brown W.J., Ford J.H., Burton N.W., Marshall A.L., Dobson A.J. (2005). Prospective Study of Physical Activity and Depressive Symptoms in Middle-Aged Women. Am. J. Prev. Med..

[31] Groce N.E. (1997). Women with Disabilities in the Developing World: Arenas for Policy Revision and Programmatic Change. J. Disabil. Policy Stud..

[32] Lau J.T.F., Griffiths S., Choi K.C., Tsui H.Y. (2010). Avoidance behaviors and negative psychological responses in the general population in the initial stage of the H1N1 pandemic in Hong Kong. BMC Infect. Dis..

[33] Elwan A. (1999). Poverty and Disability: A Survey of the Literature. Soc. Prot. Discuss. Pap..

[34] Carmody S., Murray A., Borodina M., Gouttebarge V., Massey A. (2020). When can professional sport recommence safely during the COVID-19 pandemic? Risk assessment and factors to consider. Br. J. Sports Med..

